# Organ-Based Response to Exercise in Type 1 Diabetes

**DOI:** 10.5402/2012/318194

**Published:** 2012-12-02

**Authors:** Lisa Stehno-Bittel

**Affiliations:** Department of Physical Therapy and Rehabilitation Science, University of Kansas Medical Center, Kansas City, KS 66160, USA

## Abstract

While significant research has clearly identified sedentary behavior as a risk factor for type 2 diabetes and its subsequent complications, the concept that inactivity could be linked to the complications associated with type 1 diabetes (T1D) remains underappreciated. This paper summarizes the known effects of exercise on T1D at the tissue level and focuses on the pancreas, bone, the cardiovascular system, the kidneys, skeletal muscle, and nerves. When possible, the molecular mechanisms underlying the benefits of exercise for T1D are elucidated. The general benefits of increased activity on health and the barriers to increased exercise specific to people with T1D are discussed.

## 1. Introduction

Type 1 diabetes mellitus (T1D) is the classification of all cases of diabetes that are primarily due to pancreatic beta-cell autoimmune destruction. Although there are a number of antibodies that can be detected in persons with T1D, they all result in a specific loss of pancreatic beta cells [1]. These are the insulin-producing cells of the body, so once their numbers have dwindled below a threshold level, the body loses the ability to regulate blood glucose levels, and diabetes ensues. While T1D comprises only a small percentage (5–15%) of the total cases of diabetes, it is estimated that globally 33 million people have the disease [2]. In addition, the incidence of T1D is increasing by 3–5% annual, perhaps due to unidentified environmental factors [3–5]. 

Complications of T1D are numerous, and frequently they are the basis for significant loss of function and declining quality of life. The early age of onset of this disease (average age at diagnosis is 14 years of age) results in a lifetime of exposure to erratic blood glucose levels and an increased risk of complications [6]. The most common complications are due to microvascular damage that initiates downstream destruction to organs and other tissues. In general, these microvascular-mediated complications damage the retina, kidneys, peripheral nerves and other organs. Diabetes-induced damage to the larger arteries and arterioles can result in dysfunction of the heart and brain.

Physical activity or exercise reduces the risk of most chronic diseases including vascular disease, osteoporosis, some cancers, and neurological dysfunction such as Alzheimer's disease [7]. With diabetes, there is a strong link between sedentary activity and the disease, but mainly in relationship to type 2 diabetes mellitus (T2D). In T1D, far fewer reports have focused on the role of exercise, but more recent studies suggest that exercise improves blood glucose regulation, reduces the daily insulin dosage and decreases the risk of diabetes-associated complications in people with T1D [8, 9]. Due to the paucity of research focused on exercise and T1D, most guidelines for exercise training for people with T1D are based on data obtained from nondiabetics or people with T2D [10]. In fact, between 1971 and 2011, there were only 48 randomized clinical studies focused on people with T1D and the effects of exercise [10]. From those studies the authors of a meta-analysis concluded that physical activity improved fitness, decreased insulin requirements, improved lipid levels, and vascular endothelial function in people with T1D [10]. 

The goal of this review is to summarize recent evidence concerning the benefits and hazards of exercise for people with T1D, directed at the organ and tissue level. While recent reviews have focused on clinical trials, of which there are few, relevant animal studies on the topic will be summarized in this paper.

## 2. The Pancreas

There is clear evidence that aerobic exercise decreases the amount of required insulin to maintain glycemic control in people with T1D. In fact, only 6 weeks of regular biking dropped mean insulin requirements by up to 15%, while hemoglobin A1c (HbA1c) levels were unchanged [11, 12]. In one large study of more than 19,000 people with T1D, there was a strong decrease in HbA1c levels with increased physical activity levels [13].

There are two general sites where exercise might have a direct effect on blood glucose regulation: (1) insulin secretion in the pancreatic islets; (2) insulin-stimulated glucose uptake in the skeletal muscle [14, 15]. The effects of exercise on glucose uptake in skeletal muscle will be summarized later in this paper. Here the effects of exercise on islet health and function will be critiqued.

**Figure 1:**
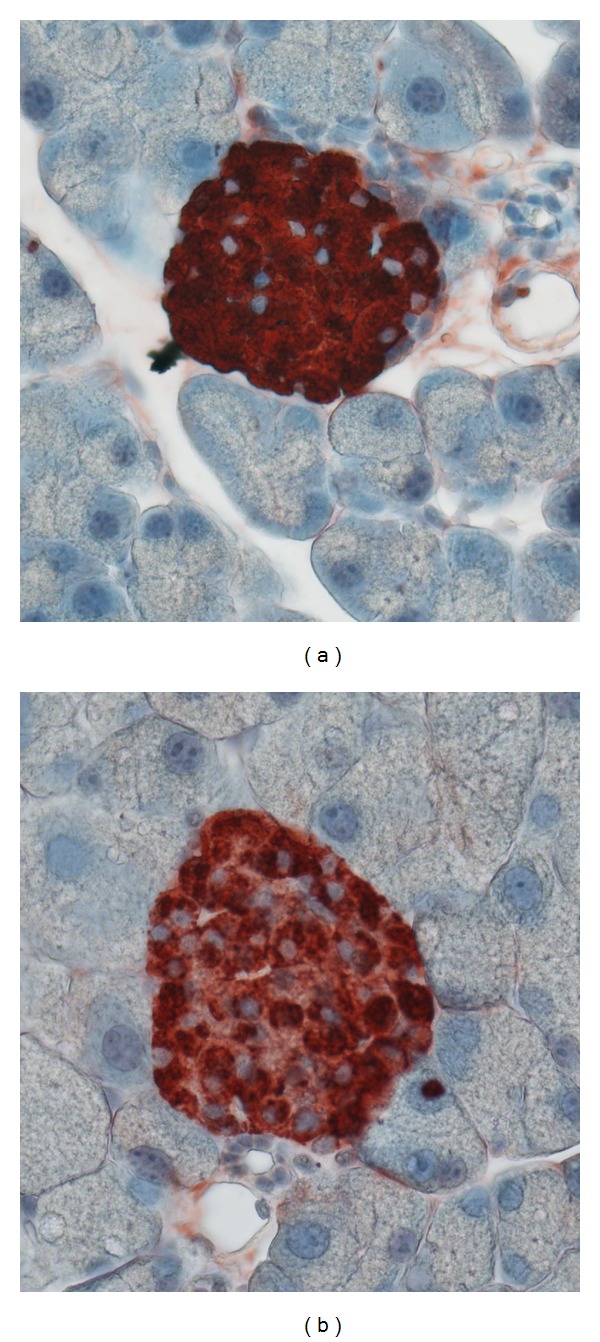
(a) A pancreatic section from a control, nondiabetic rat illustrates intense insulin staining (red) within the beta cells of a healthy islet. (b) Images of pancreatic sections from matched diabetic rats (12 weeks old) show less staining per beta cell within the islet. Images generously provided by Dr. Lesya Novikova, University of Kansas Medical Center.

In multiple studies using animal models of diabetes, aerobic exercise had no effect on the number or size of the remaining pancreatic islets, the cell clusters containing the insulin-producing beta-cells [16, 17]. Nor was there an effect on the cell composition, the percentage of alpha, beta, and delta cells, within each islet in response to exercise. However, diabetes caused a decrease in the insulin content per beta cell, (Figure 1), which was reversed by aerobic exercise. The increased insulin content per beta cell translated into more insulin per islet in the exercised group along with greater insulin secretion in response to glucose [17]. While these changes within the pancreas were significant, they were not sufficient to statistically reduce blood glucose levels [18], leading to the suggestion that exercise training may have a protective effect against the damage of oxidative stress on beta-cells [18, 19]. Thus, at a cellular level insulin content and secretion improved in diabetic animals after exercise, but it was not enough to change the whole animal glucose profile. Ways to scale-up the small cellular improvements to a level that would result in normoglycemia should be areas of vigorous research.

Recently, an important paper provided some insight into a possible mechanism linking exercise with improvements in pancreatic function [20]. Ellingsgaard et al. found that exercise stimulated muscles to produce interleukin-6 (IL-6), thus increasing systemic IL-6 levels, and resulting in a downstream increase in systemic glucagon-like peptide (GLP-1) levels [20]. This increase in GLP-1 was dependent on glucose, so that when glucose levels increased, the GLP-1 release was greater. In nondiabetic animals, the exercise-induced IL-6 release had little effect on pancreatic insulin secretion, but it prepared beta-cells for an upcoming meal resulting in a potentiating effect on insulin secretion. In other words, the IL-6 induced GLP-1 release that occurred during exercise promoted improved insulin secretion during subsequent meals. Such an effect had been previously postulated by others [19], but not proven until last year. While this study offers an important hypothesis for the molecular mechanism of exercise induced pancreatic changes, it was conducted in nondiabetic animals. Testing in a diabetic animal model could results in even greater postprandial glucose levels, because of the potentiation by diabetic animal's hyperglycemia. 

Unfortunately, there are no human clinical studies directly measuring the effect of exercise on the health and function of islets. Thus, one is left with the general hypothesis that fitness and activity can improve islet function based solely on animal studies. 

## 3. Bone

Loss of bone density is a consistent finding in children and adults with T1D [21, 22]. In general, bone turnover is reduced in this population, even when blood glucose levels are well controlled [23, 24]. The bone loss appears to be worse when diabetes is diagnosed in childhood during the growth phase of bone development. The diminished bone mineral density in children with T1D is likely because of a deficiency in insulin and insulin-like growth factor during key high growth periods [22, 25]. 

Bone mineral density loss increases with the duration of diabetes, especially in females [26]. The bone density loss puts adults at a significant risk for osteoporosis and subsequent fractures [27, 28]. In fact, a survey of over 32,000 postmenopausal women revealed that those with T1D were 12 times more likely to have hip fractures than age-matched nondiabetic women [29]. However, it is important to note that T1D also increases the risk for men to develop osteoporosis [21]. 

Bone loss associated with T1D has been identified in a variety of animal models. In mice, T1D was associated with reduced femur length, thinner growth plates, and decreased collagen expression whether the diabetes was induced chemically or genetically [30]. In diabetic rats, T1DM was associated with biomechanical changes in the long bones including a 37% reduction in the maximum load, or breaking strength, of the femur, with a 38% increase in bending stiffness [31]. These changes lead to more brittle bones with a greater risk for fractures. 

The data suggesting that tight glycemic control is important in maintaining bone density in adults [32, 33] are conflicting. Specifically, premenopausal women (mean age 34.5 years) with T1D in good glycemic control had no loss of bone density of the phalangeal bones compared to nondiabetic women [34]. In contrast, women with poor glycemic control had lower bone formation and increased bone resorption, although the differences were not statistically significant. Further, rat studies showed that insulin treatment to maintain blood glucose was ineffective in restoring the biomechanical deterioration associated with T1D bone loss [35]. 

Due to the strong benefit of moderate exercise on osteoporosis in nondiabetic populations, suggestions of exercise in the T1D population have been made [36–38], but the evidence to support the view is scant. Only one major study examined the effect of exercise training on bone mineral density in children with T1D. Importantly, 9 months of regular weight bearing activities of 90 minutes 3 times/week improved the total body bone mineral density as well as the density in the lumbar spine [32]. From the standpoint of bone, there appears to be little or no risk of exercise on bone formation or maintenance. Thus, it is suggested that activities that involve weight-bearing should be encouraged for those with T1D. 

## 4. Vascular System

Cardiovascular disease is the most frequent cause of death associated with T1D [39]. There is a 10-fold increase in the cardiovascular-related mortality compared to the nondiabetic population [40]. Youth with T1D demonstrate evidence of vascular disease, specifically atherosclerosis, early in life [40], in spite of improved tools for better glycemic control. In adults, coronary artery disease is 4-fold more likely in men and 8-fold more likely in women with T1D compared to the general population [41]. These statistics add up to higher risk of coronary artery disease in T1D even when compared to people with T2D, a group frequently identified with cardiovascular disease [42].

T1D is related to an increased risk of micro and macrovascular complications, and at the core of the disease process is chronic inflammation [43], initiated by long-term exposure to hyperglycemic conditions. Hyperglycemia plays a pivotal role in the etiology of diabetic vascular disease [44], including changes in signaling molecules such as vascular endothelial growth factor (VEGF) [45] and intracellular calcium regulation [46, 47]. In the large DCCT study and its follow up investigations, intensive glycemic control initiated when people were young resulted in a significant reduction in major vascular events nearly 20 years later [48]. When diabetics have vascular events, they are associated with an early increase in the carotid artery intima-media thickness noted even in children with T1D [49]. With aging, this increased intimal thickening resulted in decreased vessel compliance [50]. 

In animal studies, structural changes within the vessels occur early in the onset of T1D, and they include increased extracellular matrix proteins in the muscular layer of vessels and an increased incidence of damaged mitochondria in the vascular cells [51]. These differences correlated to diminished matrix metalloprotease activity in the aorta of the diabetic animals. The metalloproteases are responsible for breaking down extracellular matrix molecules [51]. Such biochemical changes in vessels likely lead to the increased intimal thickening noted in humans.

**Figure 2:**
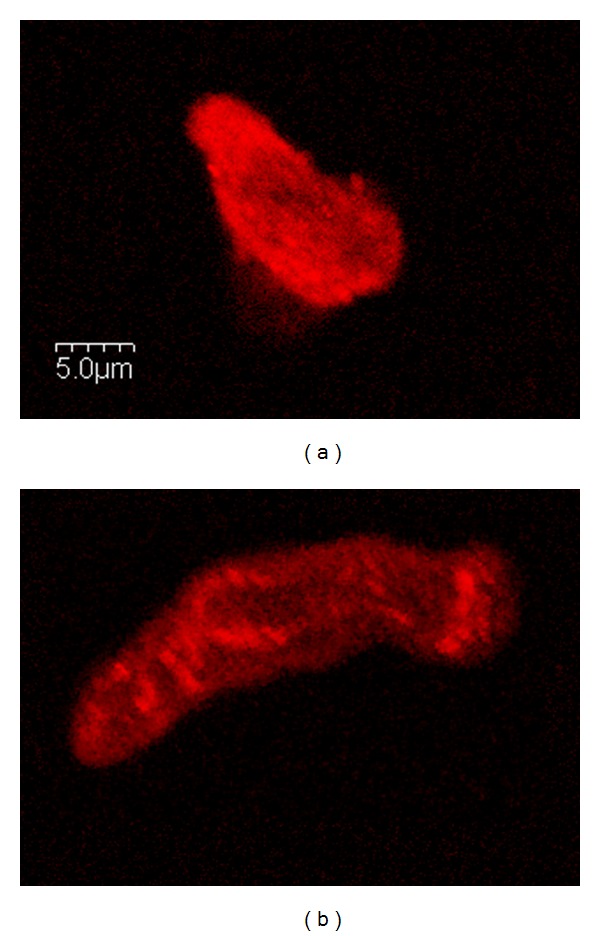
(a) Immunofluorescence staining for the calcium-pump, SERCA2, illustrates high levels of the protein distributed throughout the cell from a healthy nondiabetic animal. (b) In an age-matched, immune-mediated diabetic animal (7 weeks of diabetes), the SERCA2 levels were lower in the cell, and the SERCA distribution was punctate with a strong perinuclear localization. Scale bar = 5 *μ*m. Images generously provided by Dr. Yvonne Searls, University of Kansas Medical Center.

Intracellular calcium signaling has been altered by T1D in the vascular smooth muscle cells lining the large vessels. In response to physiological agonists, calcium transients were depressed in vascular smooth muscle cells from diabetic animals. Research in animal models and in cell culture demonstrated that specific intracellular calcium channels and calcium pumps were altered with T1D.  Figure 2 provides an example of the distribution of the sarcoplasmic calcium pump (SERCA2) in a vascular smooth muscle cell from a healthy rat and from an animal with immune-mediated T1D. There is a dramatic loss of protein amount and an altered distribution of the protein in cells from diabetic animals. This loss of calcium regulation proteins leads to blunted responses to agonists like antidiuretic hormone [47, 52]. The fact that cells grown in hyperglycemic conditions had the same altered activity as cells from diabetic animals suggests that high glucose levels are, at least partially, responsible for the altered cellular function. 

The role of exercise in preventing general vascular disease is well accepted. In fact, exercise reduced morbidity from vascular disease for a large study of men on insulin [53]. Blood pressure measurements were reduced along with improvements in the lipid profile in people with T1D after exercise training [54]. In a separate study, an individualized aerobic exercise program, along with intensive education, positively influenced lipid profiles and physical fitness in individuals with T1D [55]. Autonomic regulation of the vascular system was improved with 1 hour of daily moderate aerobic exercise for 18 weeks in adolescents with T1D [56]. Similar improvements in autonomic regulation were recorded in independent studies [57]. 

The long-term effects of these vascular improvements can be dramatic. Rats with T1D were divided into exercise or sedentary groups. Subsequently, they were given transient focal middle cerebral artery occlusions causing stroke-like brain injuries. In the animals that had been exercising, there was decreased ischemic brain injury following the stroke [58]. This may be due to the response to oxidative stress during an acute injury. In larger vessels, like the cerebral artery of diabetic rats, there was a return to normal levels of nitric oxide synthase and the resulting vessel dilation in response to exercise [59]. Interestingly, exercise training has been shown to prevent deterioration of cardiac function by mechanisms independent of blood glucose and total cholesterol levels in a pig model of T1D with dyslipidemia [60]. In addition, the excess extracellular matrix proteins discussed earlier, can be reversed with aerobic exercise. In fact the decreased luminal diameter and thicker vessel walls associated with diabetes were reversed with exercise [61]. 

A six month exercise program for adolescents with T1D demonstrated that the exercise improved the glycemic control, reduced dyslipidemia and decreased insulin requirements as well as reducing body mass index and waist circumference. The authors concluded that, “exercise is an indispensable component of medical treatment for patients with T1D” [62]. Similar results were noted in a personalized exercise program lasting only 2.5 months [63]. Thus, the major risk factors associated with vascular disease were diminished in T1D populations with exercise training. It is important to note that the effect of exercise training on the vascular system is relatively short-lived. Five weeks after the termination of an exercise study in rats, the beneficial effects on cutaneous microvascular responses to agonists had dissipated [64].

While exercise can have a positive impact on vascular risk factors, a sedentary lifestyle can have just the opposite effect. Sedentary behavior increased the body mass index and visceral fat, increasing the likelihood of coronary artery disease by 3.5-fold in people with T1D [65]. 

## 5. Heart 

People with T1D are 2–5 fold more likely to experience heart failure even when their glycemic levels are well controlled [66]. Diabetic cardiomyopathy plays a major role in heart disease that is independent of the problems associated with the coronary arteries discussed in the previous section [67]. Diabetic cardiomyopathy affects both the active and passive cardiac functional states [68]. Thus, people with T1D have a double risk for heart failure, first from the deleterious vascular changes within the coronary arteries, and second from the direct effect of diabetes on the cardiomyocytes. Clinically, diabetic myocardial disease presents with a reduced rate of pressure development and a reduced ejection fraction. In rodent models of diabetes, the disease is associated with significant compromises in the left ventricular pump function manifested as a decrease in stroke volume (up to 48% decrease) and ejection fraction (up to 28% loss) [69, 70]. 

Adolescents with T1D showed signs of reduced vascular reactivity and evidence of diastolic dysfunction and left ventricular hypertrophy compared to the matched nondiabetic control subjects [71]. In young adults with uncomplicated T1D, parasympathetic dysfunction was noted that was closely associated with diastolic deficits [72]. Even adolescent girls with T1D show blunted autonomic cardiac responses compared to nondiabetics, but in general their heart performance was better than age-matched girls with T2D [73]. Adding to the risk factors, females with T1D have a greater risk of cardiovascular disease than males with T1D, which is exactly the reverse of the sex differences associated with heart disease in the nondiabetic population. Part of the phenomenon may be explained by the higher levels of centrally distributed fat in girls with T1D compared to age-matched boys [74]. Within the heart itself, people with T1D have increased myocardial fatty acid utilization and oxidation with reduced glucose utilization [75]. Even people with uncomplicated T1D have impaired myocardial energetics that appears to be independent of the duration of the disease [76].

Exercise training studies in T1D rats showed the most influence on the diabetes-associated heart dysfunction on stroke volume, ejection fraction, and left ventricular output [77, 78]. At the end of 9 weeks of exercise training, high-resolution magnetic resonance imaging illustrated favorable functional changes, including prevention of the decreased end-diastolic and end-systolic volumes noted with diabetes [77]. The defects in the left ventricular systolic flow velocity, acceleration, and jerk associated with sedentary diabetes were all restored toward control levels in the exercise trained diabetic animals [77]. 

Most interestingly, even low intensity exercise appears to have the same effect on the heart. Diabetic rats were exercised at a low intensity so that skeletal muscle citrate synthase (a marker of training effects) levels were not altered. Even with such low intensity exercise there was still a significant decrease in resting and post-stress test heart rates and improved EKG recovery after a treadmill stress test [79].

An accumulation of interstitial collagen is one of the hallmark features of diabetic cardiomyopathy [68], resulting in increased tissue stiffness [80]. Exercise training has been shown to decrease the cardiac muscle intrinsic stiffness [77, 81]. At the ultrastructural level, there was an increase in the individual myocardial collagen fiber cross-sectional areas with diabetes, which was reversed with exercise training [82].

When considering possible mechanisms of diabetic cardiomyopathies, diacylglycerol (DAG) and its effects on protein kinase C (PKC) signaling have been implicated [83]. In a rodent model of autoimmune T1D, in which 8 weeks of treadmill exercise attenuated the diabetes-induced deterioration of left ventricular structure and function, myocardial DAG levels were decreased [84]. 

Additionally, it has been proposed that in diabetic cardiomyopathy, there is an increase in nitric oxide synthase 1 (NOS1) with a disconnection in signaling between NOS1 and the sarcoplasmic calcium channel, the ryanodine receptor. Treadmill running along with insulin therapy for T1D rats normalized the ryanodine receptor levels and improved heart function [85]. 

Structural changes in the mitochondria of cardiomyocytes have been implicated as another mechanism for diabetic heart disease [86]. Mitochondrial disruption has been quantified in rats with T1D, and these changes were also reversed by treadmill running [82]. In addition, cardiac myocytes had changes in the expression of the calcium regulating proteins. These were some of the same protein discussed earlier in the vascular section. The calcium regulating proteins were returned toward normal levels with exercise [87].

In human studies a 6 month exercise program was undertaken by nearly 150 adolescents with T1D some attending once/week and the other half attending 3 times/week [62]. The subjects in the two exercise groups had significantly lower HbA1c values, a reduced need for insulin as well as improved waist circumference. Cardiovascular risk factors were reduced including both dyslipidemia and blood pressure [62]. Furthermore, the frequency of hypoglycemic events was not statistically different between the controls and exercising groups. 

## 6. Kidney

Diabetes and hypertension are responsible for the majority of chronic kidney disorders in the USA [88]. While chronic kidney disease is present in both forms of diabetes, T1D patients tend to suffer from more diabetic nephropathy than people with T2D, because of the early onset of the disease [89]. Unfortunately, according to the US Renal Data System, there continues to be an increase in the incidence of end stage renal disease among diabetics [90]. Even in uncomplicated T1D there is a high incidence of hyperfiltration, resulting from glomerular hypertension [91]. 

 Animal studies have shown that exercise training in a type 2 model of diabetes, the obese Zucker rat, reversed the advanced glycation in the kidney and likewise ameliorated the early signs of diabetic nephropathy [92]. In animal models of T1D, improved renal function occurred with aerobic exercise in the form of a reduction of glomerular mesangial volume, attenuation of the increase in albumin excretion rate, reduction in the level of lipid peroxidation, and increased levels of antioxidants [93, 94]. In the rat model of chronic kidney disease, 11 weeks of swimming decreased fibrogenesis in the glomerular mesangium [95]. Hence it is evident that exercise targets the principal renal mechanisms thought to fail in diabetes. 

Few clinical studies on the effect of long-term exercise training on humans have focused on kidney function. However, short bouts of exercise, such as those used during exercise stress tests, are frequently utilized in assessing kidney function. During acute bouts of intensive exercise, children with T1D had higher peripheral vascular resistance during peak exercise compared to nondiabetic controls [96]. It is important to note that these children (9–19 years old) had no apparent nephropathy or systemic vasculopathy. The authors discussed the implications for kidney function. These results, and others like them, present a warning for intensive exercise in children with T1D, because of possible effects on kidney function. In fact, increases in blood pressure and albuminuria following acute exercise may be a predictive marker of later development of diabetic nephropathy [97]. However, it is important to note that albumin excretion rates increase following exercise is a normal physiological response in healthy individuals as well [98]. 

In patients with other types of chronic kidney diseases, aerobic exercise improved cardiovascular parameters including autonomic function [99]. A low-intensity exercise program for patients with a variety of chronic kidney disorders resulted in improved functional tests including the timed-up-and-go test, and a stair-climbing activity with no negative impact on kidney function [100]. Higher intensity exercise, such as intensities of 50–70 VO_2max⁡_, were also well tolerated and caused no adverse reactions or increased albumin excretion rates [101]. A recent Cochrane review of the topic analyzed 32 studies and concluded that there was evidence of significant beneficial effects of regular exercise on physical fitness, and health-related quality of life issues, but could not draw direct conclusions on kidney function [102]. This is likely because only 10 articles were published through 2012 that addressed the question and most had small subject numbers [103]. While there is insufficient information to draw a conclusion about the impact exercise on kidney function, it is hypothesized that improved cardiac function associated with exercise will positively impact kidney function. 

## 7. Skeletal Muscle

In general people with T1D have impaired activation of skeletal muscle and decreased muscular endurance during isometric contractions [104]. Crowther et al. found that the mitochondrial capacity of skeletal muscle (the maximal rate of oxidative ATP synthesis) was reduced in male patients with T1D compared with non-diabetes, and they suggested that reduced muscle mitochondrial capacity in conjunction with increased glycolytic flux represented a significant metabolic shift in T1D [105]. However, others have recently estimated that even if mitochondrial capacity were reduced, skeletal muscle still would contain sufficient mitochondria to allow a 150-fold increase in oxygen uptake per kilogram of muscle under exercise conditions [106]. Therefore, it is questionable to what extent reduced mitochondrial capacity may limit aerobic function. In a study of young women with T1D, mitochondrial capacity, muscle fiber phenotypes, and skeletal muscle capillary density were not different between diabetic and nondiabetic women [107]. While people with T1D have reduced insulin sensitivity [108], it does not appear to be due to skeletal muscle mitochondrial dysfunction [107].

If mitochondrial damage is not at the heart of the reduced insulin sensitivity and impair skeletal muscle function, then other molecular mechanisms must be involved. Cellular acetylcarnitine is a molecule involved in the regulation of fat and carbohydrate oxidation in skeletal muscle during exercise [109]. In males with T1D, exercise induced an increase in intramyocellular acetylcarnitine, which was dependent on the level of glucose control [110]. The acetylcarnitine was significantly higher in normoglycemic conditions as opposed to hyperglycemia, indicating an immediate negative effect of hyperglycemia on exercise-induced changes in muscle metabolism. Whether these changes play a role in diabetes-mediated impairment of skeletal muscle function, or whether they are a side effect of the hyperglycemic condition, is unknown at this time.

Another area of intense research in skeletal muscle function and diabetes is fatty acid metabolism. Amounts of fatty acid transporters increased following 7 days of muscle activation via exercise [111]. In prediabetic states of impaired glucose tolerance, 12 weeks of aerobic exercise decreased the triglyceride levels within the skeletal muscle cells and increased the skeletal muscle fatty acid oxidation capacity [112]. While fat oxidation rates have been shown to decrease after ingestion of high-fat diets, that trend is reversed with exercise [113].

At the heart of skeletal muscle insulin resistance is the glucose transporter protein, GLUT4 [114]. Glucose uptake into skeletal myocytes is greater during aerobic activity, as a way to support muscle contraction. Under normal conditions, insulin binds to its receptor on the skeletal muscle cells, promoting the fusion of exocytotic vesicles carrying GLUT4 to the plasma membrane. Once bound to the plasma membrane, glucose diffuses down its concentration gradient to enter the skeletal muscle cell via the GLUT4 transporter. Even a single bout of exercise can affect this pathway. For example, transcription of the GLUT4 gene increases with a single exercise session [115]. Within days of repeated exercise, GLUT4 protein levels can increase by 2-3 fold [116]. With long-term exercise (12 weeks of aerobic activity), GLUT4 expression increased dramatically [112]. The mechanisms that activate these proteins when exercising remain a very important area of research in this field. One possible mechanism centers on the signaling ion, K^+^. Skeletal muscle is also the major site of K^+^ release during exercise, and insulin reduces interstitial K^+^ by increasing muscle and liver K^+^ uptake [117]. Intensive sprint training of adults with T1D increased their skeletal muscle K^+^ regulation [118]. However, any tie between K^+^ signaling and GLUT4 transport is purely hypothetical at this time.

Thus, while the identity of the defective signaling pathway associated with diabetes may not be fully understood, or solely related to changes in GLUT4, the exercise-induced increase in GLUT4 protein levels in skeletal muscle can overcome the diabetes-induced defect, wherever it is located. Thus, a clear mechanism for the beneficial effects of exercise on skeletal muscle has been elucidated. The increase of glucose transport across the plasma membrane due to enhanced levels of GLUT4 will lower extracellular glucose levels, thereby lowering the person's blood glucose reading. In addition, when glucose is within the cell it will be better utilized in an exercise trained muscle due to upregulation of the metabolic pathways.

## 8. Nerves

Diabetic neuropathies are the most common type of neuropathies in the western world and are comprised of a number of different pathologies, including mono- and polyneuropathies [119]. Both metabolic and vascular factors are involved in the pathogenesis of diabetic neuropathy. Metabolic changes lead to oxidative stress and impaired mitochondrial function with resulting apoptosis of neurons. There are two major classifications of diabetic neuropathies, sensorimotor and autonomic. 

People with T1D and peripheral neuropathy are more likely to report injuries, especially falls, and they indicate that they feel unsafe when walking in uneven surfaces [120]. Chronic pain associated with neuropathy from either T1D or T2D decreased maximally in response to stretching exercises when muscle relaxants were also administered [121]. Interestingly, the decrease in pain was associated with improvements in sleep, indicating an improved quality of life. Perhaps most important, a 10-week aerobic and strengthening exercise program for people with T2D resulted in significant reductions in pain and neuropathic symptoms [122]. For the first time, this study quantified increased intraepidermal nerve fiber branching from proximal skin biopsies following the exercise intervention [122]. This is the first study to describe improvements in neuropathic and cutaneous nerve fiber branching following supervised exercise in people with diabetic peripheral neuropathy. The concept that nerve fiber branching could be increased with exercise in diabetics is truly game-changing. These findings are particularly promising given the short duration of the intervention, but need to be validated in a population of people with T1D.

**Figure 3:**
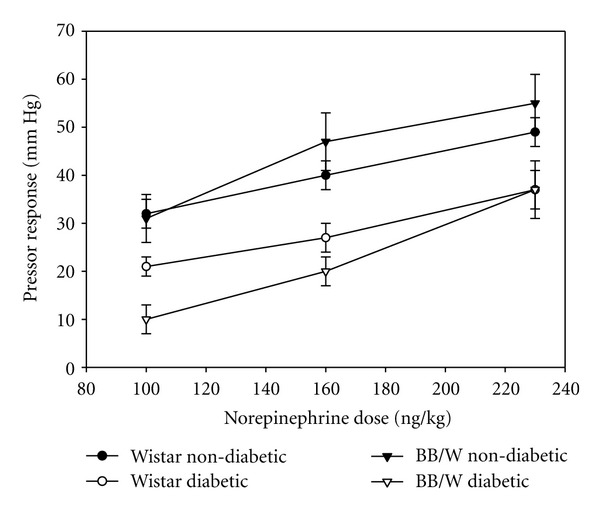
Alloxan-induced diabetic rats were pretreated with *β*-adrenergic and cholinergic blockades, and then provided graded doses of norepinephrine by intravenous injections. The change in the blood pressure elevations was plotted as the pressor response. At each norepinephrine dose, both groups of diabetic animals had blunted pressor responses compared to the matched nondiabetic controls. Figure generously provided by Dr. R. D. Bunag, University of Kansas Medical Center.

 For people with severe T1D-associated autonomic neuropathies, exercise should be undertaken with caution. During the development of exercise programs for individuals with cardiac autonomic neuropathy, the individual's tolerance to each exercise session must be monitored and evaluated. In animal studies, it is clear that T1D induces a blunted response to vasoactive substances like norepinephrine.  Figure 3 illustrates that as norepinephrine doses were increased, the pressor response was blunted in two different rat models of T1D at each dose of norepinephrine tested. Even low-intensity exercise can significantly increase heart rate variability in T1D with early cardiac autonomic neuropathy abnormalities [123]. Perhaps most alarming, individuals with severe cardiac autonomic neuropathy did not benefit from an exercise-induced increases in heart rate variability, and the variability may even put them at risk for other complications. This occurs even when it is shown that their maximal performance capacity improved [123]. These authors of this study concluded that people with T1D with cardiac autonomic neuropathy are able to tolerate low-grade exercise training and reap the benefits of exercise when initiated early in the onset of the disease process. However, exercise for people with severe cardiac autonomic neuropathies should be limited. Exercise programs for this population should be based on self-reported tolerance levels rather than standard test cut points, because of the variation in each person's response to exercise [124].

## 9. Other Tissues

 Obviously T1D, especially when chronic hyperglycemia is present, has significant impact on other tissues. While there is little research investigating the effects of exercise on those diabetes-specific changes, some studies suggest that exercise has positive effects on other organs including the eye [125] and the reproductive system [126]. However, far more research needs to be done. 

It has been suggested that general improvements in insulin sensitivity in people with T1D may be significant enough to improve symptoms of retinopathy, neuropathy, and other complications [54]. Further, there is indirect evidence suggesting that physical activity is inversely related to total mortality in T1D [127]. In adolescents, in addition to a decreased insulin requirement, physical activity improved their general health and showed a tendency towards improving mental health [128]. 

## 10. Exercise Capacity in People with T1D

In general, people with T1D have lower exercise capacity and VO_2peak_ compared to healthy age-matched subjects [73, 129, 130]. Some of these differences have been associated with the lifestyle more than the disease itself [131]. The diagnosis of diabetes, and the associated complications, can scare some parents into protecting their children by limiting moderate or high intensity activities. When activity level was correlated with VO_2peak_ in people with T1D they were highly linked [132]. In separate work, children with T1D had decreased VO_2peak_, lower peak work rates, and were more insulin insensitive compared to their matched nondiabetic counterparts [71]. Alternatively, adults with T1D who are more active have better aerobic capacity [133], and athletes with T1D have no difference in aerobic capacity compared to their nondiabetic counterparts [134]. Thus, at least some of the reduced VO_2_ capacity noted in people with T1D is likely due to lifestyle choices. However, there are also physiological changes in the ventilation function associated with T1D.

When there is a reduction in the ability to physiologically benefit from exercise in people with T1D, it may be related to poor O_2_ delivery via impaired lung diffusion capacity [135], restricted cardiac output [70], or impaired hyperemic response in the muscle [136]. However, these are thought to be dependent on the severity of the diabetic complications and the chronic blood glucose control [73, 133, 137, 138]. Thus, when the person has poor glycemic control, and more likely to develop complications, the exercise capacity is also diminished.

When investigating the cellular causes of reduced exercise tolerance several hypotheses have been put forward. One is based on the increased muscle deoxygenation at submaximal work rates noted in T1D [130]. Inefficient muscle work due to impaired peripheral vascular function or altered muscle physiology is an attractive hypothesis. However, this phenomenon is noted mainly at submaximal workloads and resolves at peak exercise levels [130]. Thus, its clinical importance is questionable. Equally intriguing is the concept that the general metabolism of people with T1D is different from nondiabetics, and thus they cannot respond to exercise in the same manner. Specifically, the response of the gluconeogenic precursors alanine and lactate to exercise was blunted in T1D [139]. It is important to note that these changes occurred after a single bout of exercise (30 min). There is no information as to whether these changes would be similar in people following chronic exercise training. 

For the past decade studies have suggested that altered sympathetic cardiovascular regulation may partially explain the reduced exercise capacity. Young people with T1D have reduced catecholamine levels during exercise, with an associated reduction in receptor sensitivity to catecholamines [140]. Further, hypoglycemic clamp experiments in people with T1D demonstrated a loss of sympathetic response to several different stimuli [141, 142]. More recently, young adults with T1D and normal weight (body mass index values) without sign of autonomic neuropathy were screened for the exercise-induced vascular response. The researchers found that those with T1D had blunted blood pressure responses, reduced capacity to increase systemic vascular resistance, and higher stroke volumes during exercise compared to healthy controls [143]. These findings led to the conclusion that cardiovascular regulation was altered in people with T1D with a reduced capacity to increase sympathetic tone with activity, described previously as diabetic cardiac autonomic neuropathy. While there may be a reduced ability to respond to exercise, these studies do not indicate that exercise is harmful, unless severe cardiac autonomic neuropathy is present.

## 11. Prescribing Exercise

In the past, many physicians were reluctant to prescribe exercise to their patients with T1D, because they did not believe it was beneficial, and/or they felt there were too many risks associated. A lack of experimental evidence on the topic supported a conservative approach toward patients that would have to live with the disease for most of their lives [98]. In addition, early reports described the impaired exercise capacity in diabetic individuals that could be made worse with exercise [144, 145], but failed to identify any of the potential positive results of an exercise intervention. The result has been a lack of understanding and under-utilization of exercise for people with T1D. As a consequence children (5–14 years old) with T1D have a lower cardiovascular fitness level than nondiabetic matched children [146]. Specifically, female children and those with poor glycemic control (high HbA1c levels) have significantly lower cardiorespiratory fitness levels [146]. Yet studies have shown that VO_2max⁡_ can increase up to 27% in people with T1D when exercise is initiated [147].

There are certainly risks for people with T1D who wish to undertake exercise programs. Obviously the extreme fluctuations in blood glucose levels must be monitored and taken into consideration when undertaking increased activity [148]. The risks of hypoglycemic events during and after exercise are real and keep many people away from regular exercise activities [149, 150]. Up to 60% of people with T1DM report the fear of hypoglycemia as the greatest deterrent to participating in exercise [151, 152]. Yet, in the few studies that reported the number of hypoglycemic events with exercise, most found no increase [13], or a minimal increase that could have been addressed through adjusted insulin dosing [10]. Careful management of the pre-exercise insulin dose and utilization of a continuous glucose monitor can greatly decrease the risk of hypoglycemia with aerobic exercise [153, 154]. The new algorithms involved in predicting glucose levels after activity and the improved monitoring tools assist people with T1D in predicting the effects of exercise on their blood glucose and making appropriate adjustments in advance [151, 155, 156]. A large meta-analysis study concluded that, at the exercise levels currently recommended by the major diabetes associations, exercise is a safe intervention with multiple benefits [10].

## 12. Summary

 Regular aerobic exercise should be the cornerstone of disease management for all people with T1D. The level of autonomic neuropathy should be assessed prior to undertaking a new exercise program. In general, dramatic benefits are noted at the cellular and tissue level that decrease mortality and reduce the risk of serious complications of the disease.
